# Safety, pharmacokinetics, and preliminary efficacy of the PARP inhibitor talazoparib in Japanese patients with advanced solid tumors: phase 1 study

**DOI:** 10.1007/s10637-021-01120-7

**Published:** 2021-06-23

**Authors:** Yoichi Naito, Yasutoshi Kuboki, Masafumi Ikeda, Kenichi Harano, Nobuaki Matsubara, Shigeyuki Toyoizumi, Yuko Mori, Natsuki Hori, Takashi Nagasawa, Takahiro Kogawa

**Affiliations:** 1 National Cancer Center Hospital East, 6-5-1, Kashiwanoha, Kashiwa, Chiba, 277-8577 Japan; 2 Pfizer R&D Japan, Shinjuku Bunka Quint Bldg. 3-22-7, Yoyogi, Shibuya-ku, Tokyo, 151-0053 Japan

**Keywords:** PARP inhibitor, Pharmacokinetics, Phase 1, Safety, Talazoparib

## Abstract

*Background*: Talazoparib is a poly(ADP-ribose) polymerase enzyme inhibitor. This open-label, non-randomized, phase 1 study of talazoparib investigated the safety, pharmacokinetics, and preliminary antitumor activity in Japanese patients with locally advanced or metastatic solid tumors, regardless of mutations in DNA damage repair-related genes, who are resistant to/ineligible for standard therapies. *Methods*: Patients received talazoparib dosed orally at 0.75 or 1 mg once daily using a modified 3 + 3 dose-escalation scheme. Primary endpoint was dose-limiting toxicities during the first cycle of talazoparib. *Results*: Nine patients (median age 62.0 years) were included: 3 and 6 patients at the 0.75 and 1.0 mg once-daily dose levels, respectively. No dose-limiting toxicities were reported. The most commonly reported treatment-emergent adverse events (≥2 patients) were anemia, stomatitis, maculopapular rash, platelet count decreased, neutrophil count decreased, and alanine aminotransferase increased. Three patients had grade ≥ 3 treatment-emergent adverse events (anemia, brain metastases [1 patient each], and neutrophil and white blood cell count decreased [same patient]). Two patients temporarily discontinued treatment due to a treatment-emergent adverse event, and 1 patient required a dose reduction for neutrophil count decreased (all at 1 mg once daily). Talazoparib exposure (C_max_ and AUC) after single and multiple dosing was slightly higher proportionally with talazoparib 1 mg than talazoparib 0.75 mg. The overall disease control rate was 44.4%, including 2 patients with stable disease. The recommended phase 2 dose of talazoparib was established as 1 mg once daily. *Conclusions*: Single-agent talazoparib was well tolerated and had preliminary antitumor activity in Japanese patients with advanced solid tumors. ClinicalTrials.gov identifier: NCT03343054 (November 17, 2017).

**Supplementary Information:**

The online version contains supplementary material available at 10.1007/s10637-021-01120-7.

## Introduction

Genomic instability is a common feature of many types of solid tumors [1], and is widely acknowledged as a key driver of carcinogenesis and acquired drug resistance [2]. Functional deficits in DNA damage repair (DDR) genes, such as breast cancer susceptibility gene (*BRCA) 1* and *BRCA2,* have been linked to genomic instability [3, 4], and individuals with germline mutations in *BRCA1/2* are at a higher risk of developing several types of tumors including breast, ovarian, prostate, and pancreatic cancer [5]. Cancer cells with DDR gene mutations rely on poly(ADP-ribose) polymerase (PARP) enzymes for DNA repair and are highly sensitive to blockade of DNA repair mechanisms by PARP inhibitors [1]. Inhibition of PARP is synthetically lethal in cells with homozygous deletions, deleterious mutations, or both in DDR genes including *BRCA1/2*, *ATM*, *PALB2*, and the *FANC* gene family [1, 3, 6, 7]. The PARP inhibitor talazoparib [8] both inhibits the PARP enzyme and effectively traps PARP on DNA, thereby preventing DNA replication and transcription as well as generating double-strand DNA breaks, leading to cell death [9, 10]. Preclinical models have indicated that trapping PARP on DNA may be more effective in inducing cancer cell death than enzymatic inhibition alone [8, 11, 12]. Although the half-maximal inhibitory concentration of talazoparib (4–11 nM) is similar to that of other PARP inhibitors currently under investigation or approved in Japan [12, 13], talazoparib has a PARP-trapping potential that is approximately 100 times greater [12]. Furthermore, although PARP inhibitors have a common class effect toxicity profile such as hematologic toxicity, fatigue, nausea, and vomiting, individual PARP inhibitors have a unique safety profile [14].

Talazoparib (oral 1 mg, once daily [QD]) is approved in the US, EU, and other countries as monotherapy for the treatment of patients with human epidermal growth factor receptor 2 (HER2)-negative advanced breast cancer with a germline *BRCA1/2* mutation [15, 16], and is under investigation in other tumor types [17, 18]. Findings from the phase 3 EMBRACA trial demonstrated that treatment with talazoparib 1 mg QD significantly improved progression-free survival (hazard ratio [HR] 0.54 [95% confidence interval (CI)] 0.41 to 0.71; *P* < 0.001) and patient-reported outcomes compared with chemotherapy in patients with HER2-negative advanced breast cancer and a germline *BRCA1/2* mutation [19, 20].

At the time of initiation of the current study, there was no clinical experience with talazoparib in Japanese patients; therefore, the objective of this phase 1 dose-escalation study was to evaluate the safety, pharmacokinetic (PK) profile, and preliminary efficacy of single-agent talazoparib in Japanese patients with advanced solid tumors, regardless of germline and/or somatic mutation status in DDR-related genes.

## Materials and methods

### Study design

This is an ongoing open-label, non-randomized, phase 1 study of the PARP inhibitor, talazoparib, in Japanese patients with locally advanced or metastatic solid tumors regardless of mutations in DDR-related genes who are resistant to standard therapies or for whom no standard therapy is available (ClinicalTrials.gov NCT03343054). The study comprises a dose-escalation phase and a dose-expansion phase conducted in 8 Japanese centers (Supplementary Fig. 1). The objective of the initial dose-escalation phase was to investigate the safety, PK (single-dose and steady-state), and preliminary antitumor activity of single-agent talazoparib in sequential cohorts of patients with advanced solid malignancies. A modified 3 + 3 dose-escalation scheme was used to determine the recommended phase 2 dose in Japanese patients; up to 3 patients could be enrolled simultaneously in a cohort although sometimes due to logistical or clinical reasons more than 3 but no more than 9 patients were enrolled at each dose level. Successive cohorts of patients received escalating doses of talazoparib on an outpatient basis starting from 0.75 mg/day. To understand the single-dose safety and single-dose PK assessments of talazoparib, a lead-in period preceding the continuous daily doses was included. Data for the dose-escalation phase are reported here (data cut-off January 2020).

The ongoing dose-expansion phase is being conducted to investigate the safety, PK, and efficacy of talazoparib, at the recommended phase 2 dose determined in the dose-escalation phase, in patients with locally advanced or metastatic breast cancer who have deleterious or suspected deleterious germline *BRCA1* or *BRCA2* mutations, previously treated with ≤3 prior chemotherapy-inclusive regimens for locally advanced or metastatic disease (Supplementary Fig. 1). Results for the dose-expansion phase will be reported in the future.

### Patients

For inclusion in the dose-escalation phase, Japanese patients (aged ≥20 years) were required to have a histologically or cytologically confirmed locally advanced or metastatic solid malignancy, which is resistant to standard therapy or for which no standard therapy was available, and an Eastern Cooperative Oncology Group (ECOG) performance status (PS) of 0 or 1. Patients who received prior platinum in any setting were not excluded.

Patients were also required to have adequate renal, liver, and bone marrow function. Samples were not collected for biomarker analysis and therefore patients were not selected based on DDR gene mutations. Full eligibility criteria are listed in the Supplementary Information.

Patients were excluded if they had symptomatic brain metastases requiring corticosteroid treatment; had undergone major surgery; had received radiation therapy, systemic cytotoxic therapies, or immunomodulators within 4 weeks of the first dose of study medication; had received previous high-dose chemotherapy requiring stem cell rescue; had been treated with prior irradiation to >25% of the bone marrow; or had received strong P-glycoprotein inhibitors/inducers or strong inhibitors of breast cancer resistance protein during the study, or within 1 week or 5 half-lives of the first study dose (whichever was longer) (see Supplementary Information for full exclusion criteria).

### Treatment

Eligible patients were assigned to one of two talazoparib treatment groups (dose levels). Successive cohorts of patients received talazoparib dosed orally at 0.75 mg QD (3 capsules of 0.25 mg) or 1 mg QD (4 capsules of 0.25 mg), with or without food, on an outpatient basis according to a modified 3 + 3 dose-escalation scheme. On Day −7 (lead-in visit), patients received a single dose of talazoparib, followed by continuous daily dosing from Day 1 of each 28-day cycle. Patients continued to receive talazoparib until either disease progression, patient refusal of study treatment, or unacceptable toxicity. Dose modifications were allowed depending on the type and severity of the toxicity encountered. To continue talazoparib, patients were required to have adequate bone marrow functions (hemoglobin ≥8.0 g/dL, absolute neutrophil count ≥1000/mm^3^, platelets ≥50,000/mm^3^). If grade 1 or grade 2 treatment-related toxicity occurred (other than renal impairment), dose interruption or dose reduction were not required. Daily dosing was paused for grade ≥3 adverse events (AEs) considered related to talazoparib. Supportive care including blood products for hematologic toxicities was allowed as appropriate per local guidance. Talazoparib dosing could resume at the next lower dose level (e.g., from 1.0 mg/day to 0.75 mg/day; 0.75 mg/day to 0.5 mg/day; 0.5 mg/day to 0.25 mg/day) when toxicity resolved to grade 1 or returned to baseline.

### Study assessments and endpoints

The primary endpoint of the dose-escalation phase was dose-limiting toxicities (DLTs) during the first cycle of talazoparib. DLTs were defined as grade ≥3 clinically significant, non-hematologic treatment-emergent adverse events ([TEAEs] excluding nausea, vomiting, or diarrhea that responded to medical intervention within 72 h, and fatigue that improved to grade ≤2 in <7 days); hematologic TEAEs (grade 4 neutropenia lasting >7 days; febrile neutropenia; grade ≥3 neutropenic infection; grade 4 thrombocytopenia; grade 4 anemia; grade 3 anemia requiring transfusion; grade ≥3 thrombocytopenia with grade ≥2 hemorrhage or requiring transfusion; interruption of daily dosing for ≥7 total days in the first cycle owing to grade 3 neutropenia or thrombocytopenia); or laboratory abnormalities (including alanine or aspartate aminotransferase [ALT; AST] >5 times the upper limit of normal [ULN], with a lower threshold if accompanied by symptoms/signs of hepatitis, plus >2 times increase above baseline values; ALT or AST ≥3 times ULN plus total bilirubin >2 times ULN; total bilirubin >5 times ULN); or failure to receive ≥75% of scheduled doses in the first cycle owing to toxicities attributable to talazoparib.

Secondary endpoints were AEs as characterized by type, frequency, severity, seriousness, and relationship to talazoparib; laboratory abnormalities; vital signs; PK parameters; and objective response status. Treatment-emergent safety data were collected throughout the study until ≥28 days after the last dose of talazoparib. AEs were coded using the Medical Dictionary for Regulatory Activities (MedDRA) Version 22.1, and the severity of AEs was graded according to Common Terminology Criteria for Adverse Events (CTCAE) Version 4.03.

PK parameters following single- and multiple-dose administration of single-agent talazoparib, included talazoparib area under the plasma concentration–time curve from 0 h to infinity (AUC_inf_; single dose) and during the dosing interval (AUC_tau_, multiple dosing); maximum observed plasma concentration (C_max_); lowest plasma concentration observed during the dosing interval (C_min_); elimination half-life (t_1/2_); time to C_max_ (T_max_); ratio of AUC_tau_ after multiple doses/AUC_tau_ after single dose (R_ac_); and ratio of AUC_tau_ after multiple doses/AUC_inf_ after single dose (R_ss_). PK parameters were calculated for each patient using non-compartmental analysis of plasma concentration–time data. Blood samples were collected pre-dose on Day −7, and on Days 1, 15, and 22 of Cycle 1, and Day 1 of Cycles 3 and 4. Post-dose blood samples were also collected at 0.5, 0.75, 1, 2, 3, 4, 6, 8, 10, and 24 h (Day −7 and Day 22 of Cycle 1 only) and at 48, 72, and 96 h (Day −7 only).

Antitumor activity was evaluated using radiological tumor assessments of all known or suspected disease sites. Imaging could include chest, abdomen, and pelvis computed tomography (CT) or magnetic resonance imaging (MRI) scans. Brain scans and bone scans were performed at baseline if disease was suspected and during the study as appropriate. CT or MRI scans were conducted at baseline (screening visit), on Day 1 (±7 days) of Cycle 2, and every other cycle thereafter, whenever disease progression was suspected, and at the time of withdrawal from treatment (if not performed within 6 weeks). Tumor response (unconfirmed) was assessed using Response Evaluation Criteria in Solid Tumors (RECIST) v.1.1, and included best overall response (complete response [CR], partial response [PR], stable disease [SD], progressive disease [PD], and non-CR/non-PD), objective response, defined as the sum of CR and PR, and overall disease control rate, defined as the sum of CR, PR, SD, and non-CR/non-PD.

### Statistical analysis

The occurrence of DLTs observed in the dose level groups (primary endpoint) was used to estimate the recommended phase 2 dose of talazoparib in patients with locally advanced or metastatic solid malignancies. According to the modified 3 + 3 design, the number of patients enrolled in the study overall and allocated to each dose level was dependent on the observed safety profile. The maximum sample size was estimated to be 18 patients. Populations included in this analysis were the safety population, comprising all patients who received ≥1 treatment with talazoparib, and the PK population, which included all enrolled patients for whom ≥1 PK parameter of interest was calculated. PK parameters and safety data were summarized descriptively.

## Results

### Patients

A total of 9 patients with a variety of solid tumor types were enrolled and treated with talazoparib in the dose-escalation phase between November 2017 and June 2019: 3 patients were treated at the 0.75 mg QD dose level and 6 patients were treated at the 1 mg QD dose level. Talazoparib was discontinued owing to disease progression in 2 (66.7%) and 6 (100.0%) patients treated at the talazoparib 0.75 mg QD and 1 mg QD dose levels, respectively. One (33.3%) patient in the talazoparib 0.75 mg QD dose level discontinued owing to a deterioration of general condition. Overall, a median (range) of 3 (1–9) treatment cycles was initiated, with the majority of patients (66.6%) initiating ≥3 cycles of talazoparib (mean [standard deviation] cumulative dose, 80.4 [44.3] mg). All 9 patients met the criteria for inclusion in the safety and PK analysis populations.

Baseline demographic and disease characteristics showed that the median (range) age was 62.0 (35–77) years, approximately half of patients were female, and a variety of solid tumor types were included (Table 1). The majority of patients had an ECOG PS of 0, and had received ≥3 systemic treatment regimens, including hormone therapy, targeted therapy, and chemotherapy.

**Table 1:** Patient demographics and baseline clinical characteristics (safety population)

	Talazoparib 0.75 mg QD *N* = 3	Talazoparib 1 mg QD *N* = 6	Total *N* = 9
Age (years), median (range)	62.0 (35–71)	63.5 (55–77)	62.0 (35–77)
Female, *n* (%)	2 (66.7)	3 (50.0)	5 (55.6)
BMI (kg/m^2^), median (range)	19.1 (17.6–22.9)	23.5 (16.4–25.3)	22.9 (16.4–25.3)
Primary cancer site, *n* (%)^a^			
Breast	1 (33.3)	0	1 (11.1)
Endometrial	0	1 (16.7)	1 (11.1)
Gastrointestinal stromal	0	1 (16.7)	1 (11.1)
Malignant melanoma	0	1 (16.7)	1 (11.1)
Pancreatic	0	1 (16.7)	1 (11.1)
Prostate	1 (33.3)	2 (33.3)	3 (33.3)
Small intestine	1 (33.3)	0	1 (11.1)
Number of prior systemic therapies, *n* (%)^b^			
0	1 (33.3)	0	1 (11.1)
1	0	1 (16.7)	1 (11.1)
2	1 (33.3)	1 (16.7)	2 (22.2)
3	1 (33.3)	0	1 (11.1)
≥4	0	4 (66.7)	4 (44.4)
ECOG Performance Status, *n* (%)			
0	3 (100.0)	5 (83.3)	8 (88.9)
1	0	1 (16.7)	1 (11.1)

^a^Locally advanced or metastatic solid tumors, unselected for germline and/or somatic mutations in DDR-related genes

^b^Includes hormone therapy, targeted therapy, and chemotherapy

BMI, body mass index; ECOG, Eastern Cooperative Oncology Group; QD, once daily

### Safety

For the primary endpoint analysis, no DLTs were reported during the first cycle of talazoparib. All patients experienced ≥1 TEAE (all-causality) (Table 2), and the majority of TEAEs were mild/moderate (≤ grade 2) in severity. The most commonly reported TEAEs (regardless of grade), occurring in ≥2 patients overall, were anemia, stomatitis, maculopapular rash, platelet count decreased, neutrophil count decreased, and ALT increased.

**Table 2:** Overview of AEs (safety population)

Patients	Talazoparib 0.75 mg QD *N* = 3	Talazoparib 1 mg QD *N* = 6	Total *N* = 9
Any TEAE, *n* (%)	3 (100.0)	6 (100.0)	9 (100.0)
Treatment-related TEAE, *n* (%)	2 (66.7)	3 (50.0)	5 (55.6)
TEAE in ≥2 patients (any grade), *n* (%)			
Anemia	0	2 (33.3)	2 (22.2)
Stomatitis	0	2 (33.3)	2 (22.2)
Maculopapular rash	0	2 (33.3)	2 (22.2)
Platelet count decreased	0	2 (33.3)	2 (22.2)
Neutrophil count decreased	1 (33.3)	1 (16.7)	2 (22.2)
ALT increased	1 (33.3)	1 (16.7)	2 (22.2)
TEAE of grade ≥ 3, *n* (%)	1 (33.3)	2 (33.3)	3 (33.3)
Treatment-related TEAE of grade ≥ 3, *n* (%)	0	1 (16.7)	1 (11.1)
SAE, *n* (%)	1 (33.3)	0	1 (11.1)
TEAE leading to death, *n* (%)	0	0	0
Permanent study drug discontinuation due to TEAE, *n* (%)	0	0	0
Temporary study drug discontinuation due to TEAE, *n* (%)	0	2 (33.3)	2 (22.2)
Dose reduction due to TEAE, *n* (%)	0	1 (16.7)	1 (11.1)
DLT	0	0	0

AE, adverse event; ALT, alanine aminotransferase; DLT, dose-limiting toxicity; QD, once daily; SAE, serious adverse event; TEAE, treatment-emergent adverse event

In total, 3 patients (33.3%) experienced 4 TEAEs of grade 3 or higher (anemia, brain metastases, neutrophil count decreased, and white blood cell count decreased); 2 events that occurred in 1 patient in the 1 mg QD dose level were considered related to treatment (neutrophil count decreased, white blood cell count decreased). One patient (0.75 mg QD dose level) experienced a serious adverse event (SAE) of brain metastases. This patient had a primary diagnosis of prostate tumor but had a history of nasopharyngeal carcinoma that, in the judgment of the investigator, had metastasized. The brain metastases were considered by the investigator to be unrelated to treatment with talazoparib.

**Fig. 1:**
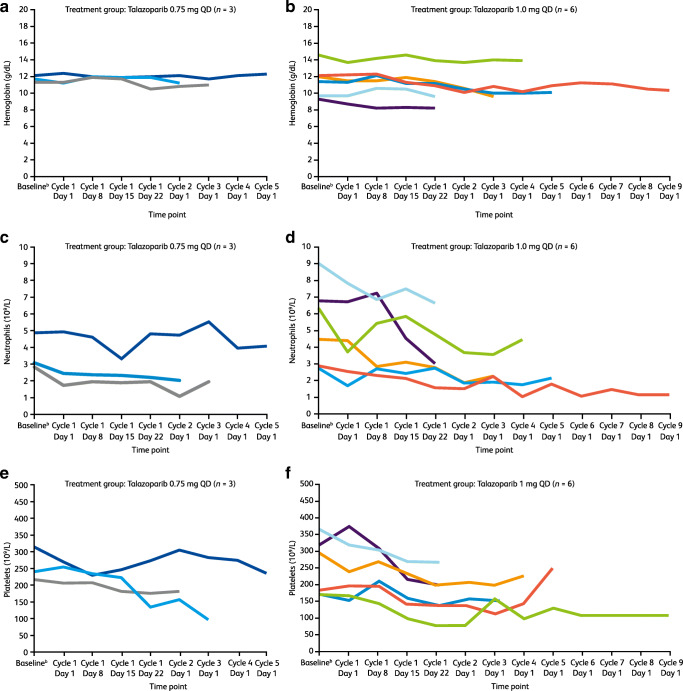
Individual laboratory values^a^ for hemoglobin (a, b), neutrophils (c, d) and platelets (e, f) during single and multiple dosing with talazoparib 0.75 mg QD (a, c, e) or 1 mg QD (b, d, f), ^a^Laboratory values are shown at scheduled visits only. Decreases in neutrophils and platelets were also observed at unscheduled visits. Patients could not start a next cycle until recovery of neutrophils to ≥1 × 10^9^/L and recovery of platelets to ≥75 × 10^9^/L; ^b^Baseline value is defined as the last value collected on or prior to the first dose date of study drug, QD, once daily

Laboratory values for hemoglobin, neutrophils, and platelets at each dose level are shown in Fig. 1a–f. Hemoglobin levels were generally maintained through the treatment period in each dose level group (Fig. 1a–b). Decreases in neutrophils (Fig. 1c–d) and platelets (Fig. 1e–f) were observed in some patients and were transient and resolved with no change to treatment or with dose interruption/reduction in most patients. Two patients in the 1 mg QD dose level temporarily discontinued treatment due to a TEAE of platelet count decreased and neutrophil count decreased (1 patient each) and a dose reduction was required by 1 patient in the 1 mg QD dose level owing to a TEAE of neutrophil count decreased. No patients permanently discontinued treatment with talazoparib due to hematologic or non-hematologic TEAEs. The recommended phase 2 dose of talazoparib was defined as 1 mg QD.

### Pharmacokinetics

Mean plasma concentrations of talazoparib rapidly reached a peak around 1 h following single or multiple dosing at 0.75 mg QD or 1 mg QD. Talazoparib was eliminated slowly with a similar pattern observed irrespective of single or multiple dosing of talazoparib at 0.75 mg QD or 1 mg QD (Supplementary Fig. 2a–c). C_max_ and AUC after single and multiple dosing with talazoparib 1 mg were slightly higher proportionally than those observed with talazoparib 0.75 mg (Table 3). Talazoparib was eliminated with a terminal phase half-life of 56.6 and 50.7 h after a single dose of 0.75 mg and 1 mg QD, respectively. Median R_ac_ and R_ss_ at the talazoparib 0.75 mg dose level were 2.8 and 1.1, respectively, and at the talazoparib 1 mg dose level were 2.3 and 1.3, respectively.

**Table 3:** Single-dose and multiple-dose plasma pharmacokinetics of talazoparib (PK population)

	Single dose		Multiple dose	
	Talazoparib 0.75 mg *N* = 3	Talazoparib 1 mg *N* = 6	Talazoparib 0.75 mg QD *N* = 3	Talazoparib 1 mg QD *N* = 6
C_max_, ng/mL	7.24 (34)	13.78 (26)	14.44 (26)	32.84 (14)
AUC_inf_, ng·h/mL	107.5 (12)	199.7 (9)	NA	NA
AUC_tau_,^a^ ng·h/mL	NA	NA	127.2 (6)	244.7 (21)
t_1/2_, h	56.60 (17.9)	50.73 (10.1)	NC	NC
T_max_, h	0.98 (0.8–1.9)	0.97 (0.5–2.0)	1.02 (1.0–1.9)	1.03 (0.7–1.9)
C_min_, ng/mL	NA	NA	2.18 (8)	3.65 (49)
R_ac_	NA	NA	2.83 (2.13–3.39)	2.32 (1.70–8.28)
R_ss_	NA	NA	1.11 (1.10–1.35)	1.30 (1.03–1.41)

Data are geometric means (geometric %CV), except mean (standard deviation) for t_½_ and median (range) for T_max_, R_ac_, and R_ss_

^a^In this study, AUC_tau_ is equivalent to AUC from time 0 to 24 h (AUC_24_)

AUC_inf_, area under the plasma concentration–time curve from 0 h to infinity; AUC_tau_, area under the plasma concentration–time curve during the dosing interval; C_max_, maximum observed plasma concentration; C_min_, lowest plasma concentration observed during the dosing interval; CV, coefficient of variation; NA, not applicable; NC, not calculated; PK, pharmacokinetic; QD, once daily; R_ac_, ratio of AUC_tau_ after multiple doses/AUC_tau_ after single dose; R_ss_, ratio of AUC_tau_ after multiple doses/AUC_inf_ after single dose; t_½_, terminal half-life; T_max_, time to C_max_

### Efficacy

Investigator-assessed antitumor responses (unconfirmed) showed that while no patients at either talazoparib dose level achieved a CR or PR, the overall disease control rate (CR + PR + SD + non-CR/non-PD) was 44.4% (Table 4). Preliminary data showed a best overall response of SD for 2 patients with prostate cancer and gastrointestinal stomal tumor, respectively, receiving talazoparib 1 mg QD and non-CR/non-PD for 1 patient with prostate cancer at each dose level.

**Table 4:** Best overall response (unconfirmed), objective response, and disease control based on derived investigator assessment (RECIST v.1.1)

	Talazoparib 0.75 mg QD *N* = 3	Talazoparib 1 mg QD *N* = 6	Total *N* = 9
Best overall response,^a^ *n* (%)			
CR	0	0	0
PR	0	0	0
SD	0	2 (33.3)	2 (22.2)
Non-CR/non-PD	1 (33.3)	1 (16.7)	2 (22.2)
PD	2 (66.7)	3 (50.0)	5 (55.6)
Objective response (CR + PR), *n* (%)	0	0	0
95% CI	0–70.8	0–45.9	0–33.6
Disease control (CR + PR + SD + non-CR/non-PD), *n* (%)	1 (33.3)	3 (50.0)	4 (44.4)
95% CI	0.8–90.6	11.8–88.2	13.7–78.8

^a^Unconfirmed

CI, confidence interval, CR, complete response; PD, progressive disease, PR, partial response; RECIST, Response Evaluation Criteria in Solid Tumors; SD, stable disease

## Discussion

The results of this phase 1 dose-escalation study demonstrated that single-agent talazoparib at the recommended phase 2 dose of 1 mg QD was well tolerated in Japanese patients when administered either as a single dose or continuously.

During the dose-escalation phase, all patients experienced at least one AE. Hematologic toxicity included anemia (2 patients at 1 mg QD), platelet count decreased (2 patients at 1 mg QD), neutrophil count decreased (1 patient at each dose level), and white blood cell count decreased (1 patient at 1 mg QD who also experienced a neutrophil count decrease at this dose level). One patient at each of the dose levels experienced increased ALT, respectively. An increase in AST was also reported in 1 patient at 1 mg QD dose level (data not shown); all ALT and AST events were grade 1 in severity. An increase in transaminase levels was also observed in a small number of patients in the dose expansion phase of the phase 1 study of talazoparib in non-Japanese patients [21]. No DLTs were reported during the first cycle of talazoparib at either dose level of 0.75 mg QD or 1 mg QD (the primary endpoint). The majority of TEAEs were mild or moderate in intensity, with SAEs and treatment-related grade ≥3 TEAEs only reported by 1 of 9 patients. For most patients, these TEAEs resolved without dose modification (reduction) or dose interruption, and no patients permanently discontinued talazoparib treatment due to hematologic TEAEs. Although some patients in this study received >3 regimens of systemic therapies prior to study participation, it should be noted that those patients were not heavily pre-treated with chemotherapy regimens (data not shown), therefore, this may account for the low level of safety events, particularly those relating to bone marrow suppression, which were observed in this study.

The safety profile of talazoparib demonstrated in this study was consistent with the findings of other clinical studies in non-Japanese patients. In a phase 1 dose-escalation study in non-Japanese patients with germline *BRCA1/2* mutations in several types of advanced cancers [9], the primary AEs related to talazoparib (dose levels 0.025 to 1.1 mg QD) were hematologic (anemia, thrombocytopenia, and neutropenia) and generally resolved with drug interruption and/or dose reduction and routine medical intervention [9]. Similarly, in the phase 3 EMBRACA study in non-Japanese patients with advanced breast cancer, the most common hematologic AEs with talazoparib were also anemia, neutropenia, and thrombocytopenia. Only 1.4% of patients discontinued from the EMBRACA study due to hematologic toxicities (0.7% due to anemia) [22]. The hematologic toxicities associated with talazoparib in non-Japanese patients were generally transient and resolved with dose modification and supportive care including growth factors and transfusions [9, 22].

The PK profile of talazoparib in Japanese patients was also generally similar to that previously observed in non-Japanese patients [9], with rapid absorption noted after oral dosing. In the phase 1 dose-escalation study of talazoparib in non-Japanese patients, dose-proportionality was observed over the dose range studied (0.025–1.1 mg QD) [9]. In the present study, drug exposure at the talazoparib 1 mg dose level was slightly higher than dose-proportional compared with the talazoparib 0.75 mg dose level; however, the C_max_ and AUC values in Japanese patients in this study were comparable with those reported previously in non-Japanese patients at similar dose levels [9].

Available efficacy results showed a disease control rate of 44.4% in patients with solid tumors with no molecular selection for DDR gene mutations. It should be noted that prior platinum therapy was permitted for study participation. These data provide preliminary evidence of the antitumor effects of talazoparib in patients with advanced solid tumors; however, further studies are required to confirm these results.

Limitations of this study include the small number of patients enrolled in the dose-escalation phase, and the open-label, non-randomized design of the study. In addition, genomic and/or germline testing were not conducted, therefore some patients may or may not have had DDR mutations that affected the study findings.

In conclusion, single-agent talazoparib 1 mg QD was well tolerated in Japanese patients with advanced solid tumors who were unselected for mutations in DDR-related genes, and had a PK profile consistent with that reported in non-Japanese patients. Single agent talazoparib 1 mg QD also demonstrated preliminary antitumor activity in Japanese patients. This trial is now expanding to evaluate the efficacy of talazoparib in Japanese patients with HER2-negative locally advanced or metastatic breast cancer and a deleterious germline *BRCA1* or *BRCA2* mutation. In addition, results of the ongoing phase 3 global clinical trial of talazoparib plus enzalutamide versus enzalutamide monotherapy in patients with metastatic castration-resistant prostate cancer (NCT03395197) will provide supportive evidence for the participation of Japanese patients in future global clinical trials.

## Supplementary Information

ESM 1 (DOCX 204 kb)

## References

[1] Javle M, Curtin NJ (2011). The potential for poly (ADP-ribose) polymerase inhibitors in cancer therapy. Ther Adv Med Oncol.

[2] Andor N, Maley CC, Ji HP (2017). Genomic instability in Cancer: teetering on the limit of tolerance. Cancer Res.

[3] Helleday T (2011). The underlying mechanism for the PARP and BRCA synthetic lethality: clearing up the misunderstandings. Mol Oncol.

[4] Venkitaraman AR (2002). Cancer susceptibility and the functions of BRCA1 and BRCA2. Cell.

[5] Mersch J, Jackson MA, Park M, Nebgen D, Peterson SK, Singletary C, Arun BK, Litton JK (2015). Cancers associated with *BRCA1* and *BRCA2* mutations other than breast and ovarian. Cancer.

[6] Ashworth A, Lord CJ (2018). Synthetic lethal therapies for cancer: what's next after PARP inhibitors?. Nat Rev Clin Oncol.

[7] Lord CJ, Ashworth A (2017). PARP inhibitors: synthetic lethality in the clinic. Science.

[8] Shen Y, Rehman FL, Feng Y, Boshuizen J, Bajrami I, Elliott R, Wang B, Lord CJ, Post LE, Ashworth A (2013) BMN 673, a novel and highly potent PARP1/2 inhibitor for the treatment of human cancers with DNA repair deficiency. Clin Cancer Res 19:5003–5015. 10.1158/1078-0432.Ccr-13-1391 10.1158/1078-0432.CCR-13-1391 PMC6485449 23881923

[9] de Bono J, Ramanathan RK, Mina L, Chugh R, Glaspy J, Rafii S, Kaye S, Sachdev J, Heymach J, Smith DC, Henshaw JW, Herriott A, Patterson M, Curtin NJ, Byers LA, Wainberg ZA (2017) Phase I, dose-escalation, two-part trial of the PARP inhibitor talazoparib in patients with advanced germline *BRCA1/2* mutations and selected sporadic cancers. Cancer Discov 7:620–629. 10.1158/2159-8290.Cd-16-1250 10.1158/2159-8290.CD-16-1250 PMC5905335 28242752

[10] Shen Y, Aoyagi-Scharber M, Wang B (2015). Trapping poly(ADP-ribose) polymerase. J Pharmacol Exp Ther.

[11] Murai J, Huang SN, Das BB, Renaud A, Zhang Y, Doroshow JH, Ji J, Takeda S, Pommier Y (2012). Trapping of PARP1 and PARP2 by clinical PARP inhibitors. Cancer Res.

[12] Murai J, Huang SY, Renaud A, Zhang Y, Ji J, Takeda S, Morris J, Teicher B, Doroshow JH, Pommier Y (2014). Stereospecific PARP trapping by BMN 673 and comparison with olaparib and rucaparib. Mol Cancer Ther.

[13] Inuzuka M, Nakamura S (2019) Hereditary breast and ovarian cancer syndrome. Gan To Kagaku Ryoho 46:1109–1113 31296812

[14] LaFargue CJ, Dal Molin GZ, Sood AK, Coleman RL (2019). Exploring and comparing adverse events between PARP inhibitors. Lancet Oncol.

[15] U.S. Food and Drug Administration (2020) TALZENNA^®^ (talazoparib) prescribing information. http://labeling.pfizer.com/ShowLabeling.aspx?id=11046. Accessed October 08, 2020

[16] European Medicines Agency (2019) TALZENNA^®^ (talazoparib) Summary of Product Characteristics. https://www.ema.europa.eu/en/documents/product-information/talzenna-epar-product-information_en.pdf. Accessed October 08, 2020

[17] de Bono J, Mehra N, Higano CS, Saad F, Buttigliero C, Mata M, Chen H-C, Healy CG, Paccagnella ML, Czibere A, Fizazi K TALAPRO-1: a phase 2 study of talazoparib (TALA) in men with DNA damage repair mutations (DDRmut) and metastatic castration-resistant prostate cancer (mCRPC) – First Interim Analysis (IA). In: Genitourinary Cancers Symposium (ASCO-GU), San Francisco, CA, February 13–15, 2020

[18] Agarwal N, Shore ND, Dunshee C, Karsh LI, Azad A, Fay AP, Carles J, Sullivan B, Di Santo N, Elmeliegy M, Lin X, Quek RGW, Czibere A, Fizazi K TALAPRO-2: a placebo-controlled phase 3 study of talazoparib (TALA) plus enzalutamide (ENZA) for patients with first-line metastatic castration-resistant prostate cancer (mCRPC). In: Genitourinary Cancers Symposium (ASCO-GU), San Francisco, CA, February 13–15, 2020

[19] Litton JK, Rugo HS, Ettl J, Hurvitz SA, Gonçalves A, Lee K-H, Fehrenbacher L, Yerushalmi R, Mina LA, Martin M, Roché H, Im Y-H, Quek RGW, Markova D, Tudor IC, Hannah AL, Eiermann W, Blum JL (2018). Talazoparib in patients with advanced breast cancer and a germline *BRCA* mutation. N Engl J Med.

[20] Ettl J, Quek RGW, Lee K-H, Rugo HS, Hurvitz S, Gonçalves A, Fehrenbacher L, Yerushalmi R, Mina LA, Martin M, Roché H, Im Y-H, Markova D, Bhattacharyya H, Hannah AL, Eiermann W, Blum JL, Litton JK (2018). Quality of life with talazoparib versus physician's choice of chemotherapy in patients with advanced breast cancer and germline *BRCA1/2* mutation: patient-reported outcomes from the EMBRACA phase III trial. Ann Oncol.

[21] ClinicalTrials.gov (2021) Study of Talazoparib, a PARP inhibitor, in patients with advanced or recurrent solid tumors (NCT01286987). ClinicalTrials.gov. https://clinicaltrials.gov/ct2/show/results/NCT01286987. Accessed 5 Mar 2021

[22] Hurvitz SA, Gonçalves A, Rugo HS, Lee K-H, Fehrenbacher L, Mina LA, Diab S, Blum JL, Chakrabarti J, Elmeliegy M, DeAnnuntis L, Gauthier E, Czibere A, Tudor IC, Quek RGW, Litton JK, Ettl J (2020) Talazoparib in patients with a germline *BRCA*-mutated advanced breast cancer: detailed safety analyses from the phase III EMBRACA trial. Oncologist 25:e439–e450. 10.1634/theoncologist.2019-0493 10.1634/theoncologist.2019-0493 PMC7066700 32162822

