# Supplementary material for: Safety, pharmacokinetics, and preliminary efficacy of the PARP inhibitor talazoparib in Japanese patients with advanced solid tumors: phase 1 study
Source: Invest New Drugs. 2021 Jun 23;39(6):1568–76. doi: 10.1007/s10637-021-01120-7 (PMC8541992; doi:10.1007/s10637-021-01120-7)

**Supplementary** **information**

**Article title: Safety, pharmacokinetics, and preliminary efficacy of talazoparib in Japanese patients with advanced solid tumors: phase 1 study**

**Journal title:** *Investigational New Drugs*

**Authors:** Yoichi Naito, Yasutoshi Kuboki, Masafumi Ikeda, Kenichi Harano, Nobuaki Matsubara, Shigeyuki Toyoizumi, Yuko Mori, Natsuki Hori, Takashi Nagasawa, Takahiro Kogawa

**Corresponding author and contact details:** Yoichi Naito, National Cancer Center Hospital East, 6-5-1, Kashiwanoha, Kashiwa, Chiba 277-8577, Japan. Email: ynaito@east.ncc.go.jp

**Supplementary Fig 1** Study design


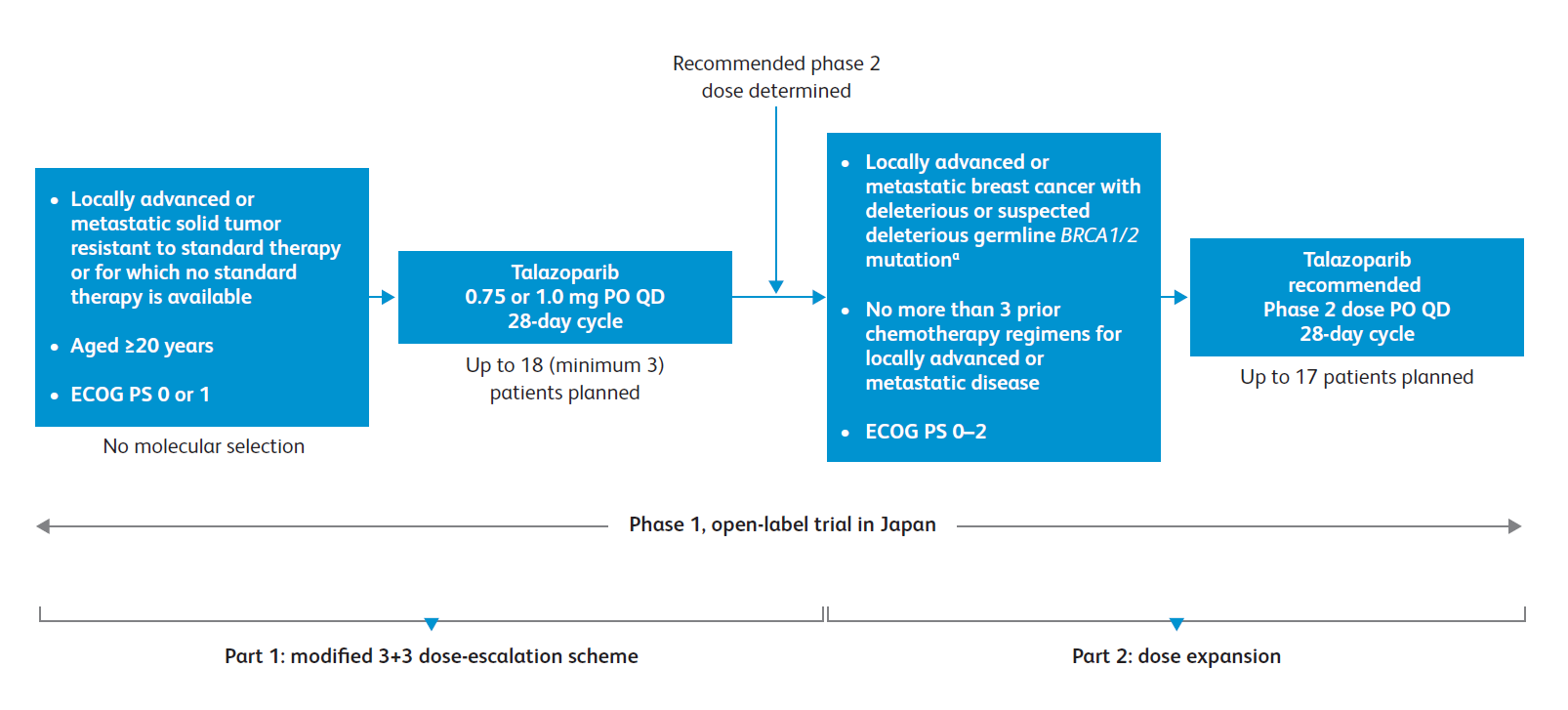


^a^Excludes HER2-positive breast cancer

*BRCA 1/2,* breast cancer susceptibility genes 1 or 2; DLT, dose-limiting toxicities; ECOG, Eastern Cooperative Oncology Group; HER2, human epidermal growth factor receptor 2; PO, per os (orally); PS, performance status; QD, once daily

**Supplementary** **Fig 2** Mean plasma concentration of talazoparib over time after (a) a single dose of talazoparib 0.75 mg or 1 mg (time from 0 to 168 hours), (b) a single dose of talazoparib 0.75 mg or 1 mg (time from 0 to 24 hours), and (c) multiple dosing with talazoparib 0.75 mg QD or 1 mg QD


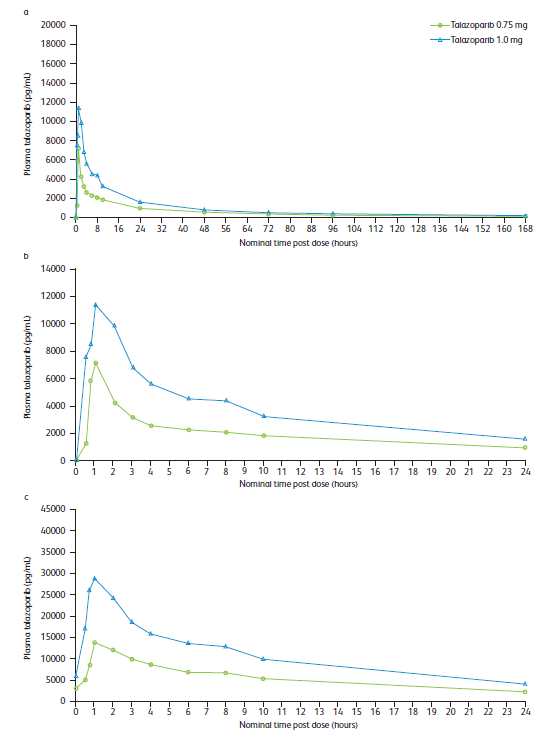


QD, once daily

**Inclusion criteria**

Patients must meet all of the following inclusion criteria to be eligible for enrollment in the study:

- Histologic or cytologic diagnosis of locally advanced or metastatic solid tumor that is resistant to standard therapy or for which no standard therapy is available.
- Females and/or male patients age ≥20 years.
- Eastern Cooperative Oncology Group (ECOG) Performance Status (PS) 0 or 1.
- Adequate bone marrow function, including:
  - Absolute neutrophil count ≥1,500/mm^3^ or ≥1.5 × 10^9^/L without the use of growth factor;
  - Platelets ≥100,000/mm^3^ or ≥100 × 10^9^/L without the use of platelet transfusions or growth factors;
  - Hemoglobin ≥9 g/dL , with last transfusion at least 14 days prior to the first dose of study treatment.
- Adequate renal function, including an estimated creatinine clearance ≥45 mL/min as calculated using the method standard for the institution.
- Adequate liver function, including:
  - Serum total bilirubin ≤1.5 × upper limit of normal (ULN) unless the patient has documented Gilbert syndrome (≤3 × ULN for Gilbert syndrome);
  - Aspartate aminotransferase (AST) and alanine aminotransferase (ALT) ≤2.5 × ULN (≤5.0 × ULN if there is liver involvement by the tumor);
  - Alkaline phosphatase ≤2.5 × ULN (≤5 × ULN in case of bone metastasis).
- Able to take oral medications.
- Resolved acute effects of any prior therapy to baseline severity or Common Terminology Criteria for Adverse Events grade ≤1 except for adverse events not constituting a safety risk by investigator judgment.
- Serum or urine pregnancy test (for females of childbearing potential) negative at screening.
- Female patients of non-childbearing potential must meet at least 1 of the following criteria:
  - Achieved postmenopausal status, defined as follows: cessation of regular menses for at least 12 consecutive months with no alternative pathological or physiological cause; and have a serum follicle-stimulating hormone level confirming the postmenopausal state;
  - Have undergone a documented hysterectomy and/or bilateral oophorectomy;
  - Have medically confirmed ovarian failure.
  - All other female patients (including female patients with tubal ligations) are considered to be of childbearing potential.
- Evidence of a personally signed and dated informed consent document indicating that the patient has been informed of all pertinent aspects of the study.
- Willing and able to comply with scheduled visits, treatment plan, laboratory tests, and other procedures.

**Exclusion criteria**

Patients with any of the following characteristics/conditions will not be included in the study:

- Patients with known symptomatic brain metastases requiring steroids. Patients with previously diagnosed brain metastases are eligible if they have completed their treatment and have recovered from the acute effects of radiation therapy or surgery prior to study entry, have discontinued corticosteroid treatment for these metastases for at least 4 weeks and are neurologically stable.
- Major surgery within 4 weeks prior to the first dose of study treatment.
- Radiation therapy within 4 weeks prior to the first dose of study treatment. Palliative radiotherapy for the treatment of painful bony lesions within 2 weeks prior to the first dose of study treatment.
- Any antitumor systemic cytotoxic therapies within 4 weeks prior to the first dose of study treatment (6 weeks for nitrosoureas or mitomycin-C), treatment with immune modulators (including, but not limited to, corticosteroids (at a prednisone-equivalent dose of >10 mg/day), cyclosporine and tacrolimus; locally active treatments such as Beconase® are allowed) within 4 weeks prior to the first dose of study treatment.
- Previous high-dose chemotherapy requiring stem cell rescue.
- Prior irradiation to >25% of the bone marrow
- Active and clinically significant bacterial, fungal, or viral infection, including hepatitis B virus, hepatitis C virus, known human immunodeficiency virus, or acquired immunodeficiency syndrome-related illness.
- Myocardial infarction within 6 months before starting therapy, symptomatic congestive heart failure (New York Heart Association class III or IV), unstable angina, or unstable cardiac arrhythmia requiring medication. Stable cardiac arrhythmia (e.g., chronic atrial fibrillation controlled by medication) can be eligible.
- Hypertension that cannot be controlled by medications (>150/100 mm Hg despite optimal medical therapy)
- Participation in other studies involving investigational drug(s) within 4 weeks prior to the first dose of study treatment.
- Known or suspected hypersensitivity to active ingredient/excipients.
- Other acute or chronic medical or psychiatric condition, including recent (within the past year) or active suicidal ideation or behavior or laboratory abnormality that may increase the risk associated with study participation or investigational product administration or may interfere with the interpretation of study results and, in the judgment of the investigator, would make the patient inappropriate for entry into this study.
- Investigator site staff members directly involved in the conduct of the study and their family members, site staff members otherwise supervised by the investigator, or patients who are Pfizer employees, including their family members, directly involved in the conduct of the study.
- Fertile male patients and female patients of childbearing potential who are unwilling or unable to use 2 highly effective methods of contraception as outlined in this protocol for the duration of the study and for 6 months (180 days) in male patients and 7 months in female patients after the last dose of investigational product.
- Breastfeeding at screening or at any time during study participation.
- Current use of a strong P-glycoprotein inhibitor (e.g., dronedarone, quinidine, ranolazine, verapamil, itraconazole, ketoconazole), strong P-glycoprotein inducer (e.g., rifampin, tipranavir, ritonavir), or strong inhibitor of BCRP within 1 week or 5 half-lives whichever is longer prior to the first dose of study treatment. Patients on verapamil are excluded from the study unless the patient and investigator agree to switch to an alternative, allowed antihypertensive. Any such switch must begin at least 7 days before the first dose of study treatment and additional blood pressure monitoring will be performed as considered medically necessary by the investigator to monitor blood pressure control.
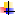

Supplement: Supplementary file 1 — (DOCX 204 kb) [file 10637_2021_1120_MOESM1_ESM.docx]
